# Combined Algorithm-Based Adaptations of Insulin Dose and Carbohydrate Intake During Exercise in Children With Type 1 Diabetes: Results From the CAR2DIAB Study

**DOI:** 10.3389/fendo.2021.658311

**Published:** 2021-08-26

**Authors:** Philippe Antoine Lysy, Hélène Absil, Emy Gasser, Hasnae Boughaleb, Thierry Barrea, Stéphane Moniotte

**Affiliations:** ^1^ Pediatric Endocrinology, Cliniques universitaires Saint-Luc, Brussels, Belgium; ^2^ Pôle PEDI, Institut de Recherche Expérimentale et Clinique, UCLouvain, Brussels, Belgium; ^3^ Pediatric Cardiology, Cliniques universitaires Saint-Luc, Brussels, Belgium

**Keywords:** type 1 diabetes, children, adolescents, exercise, CGM, treadmill, algorithm

## Abstract

**Objectives:**

To evaluate the evolution of subcutaneous glucose during two sessions of monitored aerobic exercise in children or adolescents with type 1 diabetes after adaptation of insulin doses and carbohydrate intake according to a combined algorithm.

**Methods:**

Twelve patients with type 1 diabetes (15.1 ± 2 years; diabetes duration: 9.5 ± 3.1 years) performed two series of exercise sessions after cardiac evaluation. The first series (TE#1) consisted in a monitored exercise of moderate to vigorous intensity coupled with a bout of maximum effort. The second series of exercises (TE#2) was carried out in real life during exercises categorized and monitored by connected watches. TE#2 sessions were performed after adaptation of insulin doses and fast-acting carbohydrates according to decision algorithms.

**Results:**

Patients did not experience episodes of severe hypoglycemia, symptomatic hyperglycemia, or hyperglycemia associated with ketosis. Analysis of CGM data (15 h) during TE#2 sessions revealed an overall improvement in glycemic average [± standard deviation] (104 ± 14 mg/dl *vs.* 122 ± 17 mg/dl during TE#1; *p* < 0.001), associated with a decrease in proportion of hyperglycemia in periods ranging from 4 h to 15 h after performing the exercises. The proportion of hypoglycemia was not changed, except during the TE#2 +4–8 h period, where a significant increase in hypoglycemia <60 mg/dl was observed (25% *vs.* 6.2%; *p* = 0.04), yet without concurrent complications.

**Conclusion:**

In our pediatric series, the application of algorithmic adaptations of insulin doses and carbohydrate intake has globally improved glycemic control during 15 h after real-time exercises performed by children and adolescents with type 1 diabetes.

## Introduction

Physical activity is an essential component of the treatment of diabetes mellitus (1) and its regular practice is encouraged in all forms of the disease (2). Yet, patients with type 1 diabetes (T1D) doing sports have to face various challenges to maintain blood glucose levels into the normal range. Compared to adults, children and adolescents are more prone to glycemic dysregulation during sports because of distinctive features of their exercises [e.g., unplanned activities, mixed bouts of aerobic and anaerobic phases, and difficulty to anticipate carbohydrate (CHO) intake in young patients] and the lack of uniform recommendations for insulin adaptation and CHO intake that may “fit for all” (2). The difficulty to gather scientific evidence from clinical trials derives partly from the heterogeneity of research protocols (e.g., in terms of study end points or specifics of training sessions) (3) and from the difficulty to propose standardized exercise protocols to a substantial and homogeneous cohort of patients.

Consensus guidelines are being progressively developed to improve education to patients doing sports in terms of insulin adjustment and dietary management (4), for interpretation of data from continuous glucose monitoring (CGM) (5) and for alternation of the forms and intensity of exercise to mitigate acute glucose fluctuations (6, 7). Nevertheless, there is an obvious idiosyncratic variation of glucose levels during physical activity (8) and, in the context of diabetes education, it is crucial to help patients to accumulate individual experience in peculiar forms of exercise, since the patient’s experience will feed baseline recommendations provided in consultation.

In a recent study (TREAD-DIAB trial (9), we evaluated the needs of children and adolescents in terms of insulin modifications during sports. During two rounds of exercise sessions, we applied an algorithm that helped us to precisely and individually adapt insulin administration during exercise sessions (9). Our results showed the possibility to normalize subcutaneous glucose (SG) for patients under continuous subcutaneous insulin infusion (CSII) therapy. Because this goal was more challenging to achieve in young patients under multiple daily injection (MDI) regimens, we conducted a new study (CAR2DIAB trial) with the objective to fine-tune insulin injections and CHO intake to patients regardless of their treatment regimen.

## Research Design and Methods

### Consent Procedure

The study was designed as a non-randomized controlled monocentric intervention clinical trial in children and adolescent with T1D attending outpatient clinic in a tertiary health care center (Cliniques universitaires Saint-Luc). Informed consent was obtained from the parents and assent was obtained from children after receiving adapted information. The local ethical Committee (Comité d’Ethique Hospitalo-Facultaire) approved the study protocol (2017/28MAR/164). The study was conducted in accordance to the Declaration of Helsinki.

### Study Procedures

The study consisted of two consecutive sessions of monitored exercise, the first being standardized and organized in our outpatient clinic and the second being performed in real-life settings (i.e., in the context of usual sports practiced by the patients). During the first visit (baseline), patients were evaluated for age, sex, Tanner stage; height, weight, and BMI standard deviation scores (according to Belgian reference charts (10); duration of T1D; associated diseases and general physical evaluation; and HbA_1C_ levels. On the two main meals preceding the exercise sessions (day 1: dinner; day 2: breakfast and lunch), a standardized diet was assigned to the patient based on the recommended daily intake requirements depending on age and sex (11).

All patients were under the CGM system (Freestyle Libre^®^, Abbott) and were asked to self-monitor capillary blood glucose when presenting symptoms of hypoglycemia. Patients were also asked to avoid consuming sugary drinks between meals, practicing sports or exercising during the 24 h preceding the study exercise, or consuming acetaminophen during the CGM reading. Twenty-four hours after the study exercise sessions, patients were asked to resume their usual daily activities and diet.

The first exercise (TE#1) sessions were performed with patients following their usual insulin regimen (i.e., pump therapy with Medtronic MiniMed™ 640g^®^ or multiple daily injections [MDI, consisting of four daily preprandial injections of fast-acting insulin and one daily injection of basal insulin at bedtime]) to determine spontaneous subcutaneous glucose (SG) profiles of the subjects during exercise and the 15 following hours. Patients were asked to follow instructions for CHO intake as per the algorithm proposed by Riddell and colleagues (12) during the exercise sessions and for a period of 2 h or until the next snack or meal. In brief, patients were asked to ingest 16 g of CHOs when SG levels were below 90 mg/dl, 16 g or 20 g of CHOs when SG levels were between 90 and 108 mg/dl with the sensor respectively indicating one or two downward arrows (i.e., a drop of SG levels), and 8 g of CHOs when SG levels were between 110 and 125 mg/dl. Fast-acting CHOs were provided as solutions of grenadine (Teisseire^®^) in vials containing 8, 16, or 20 g of glucose, to ensure an appropriate intake of CHO during sessions. After the first session, glucose curves, insulin administration, and potential corrections of hypoglycemia (<60 mg/dl) were integrated and analyzed *via* an algorithm (as described elsewhere (9) to propose modifications of insulin administration during a second session of appointments. These series of sessions (TE#2) were designed to assess the provisional adjustments of insulin regimen to achieve glycemic control within targets (70–180 mg/dl) during and after exercise. Based on the PAQ-C and PAQ-A questionnaires (13–15), the participants were asked to evaluate their level of fitness.

The second exercise sessions (TE#2) were performed 1 month after the first session and consisted of an exercise in real time, corresponding to sporting habits of the patient within a category [i.e., tennis (60’), football or hockey training (but not match, 60–75’), running or swimming (45–60’), etc.]. The patient was asked to follow instructions for CHO intake as described above and wear a smart watch (Garmin Forerunner^®^) to quantify the intensity of the effort. After Test#2, a second analysis of CGM data was performed to evaluate the effectiveness of our recommendations.

For each session, during 3 days starting the day before the test exercise, patients were asked to document the composition of their meals, the presence of symptoms of hypo- or hyperglycemia, and the composition (quantity and quality) of any extra CHO intake during hypoglycemia.

### Exercise Protocol

The standardized exercise protocol consisted of brisk aerobic walking on a treadmill (HP Cosmos Mercury) at a maximal level (VO_2_ max, duration 10 to 15 min) immediately followed by a rest period of 3 min, and then by a moderate-to-vigorous effort (70% of the theoretical maximum heart rate) for a total duration of 30 min (Master Screen CPX with Cardiosoft). Speed and slope of the treadmill were individually tailored to achieve heart rate goals.

An EKG and echocardiogram were performed before first exercise to rule out heart disease or contra-indications to exercise. Each moderate-to-vigorous exercise was immediately preceded and followed by lung function tests [determination of forced expiratory volume in one second (FEV1), vital capacity (VC), FEV1/forced vital capacity (FEC1/VC) (Tiffeneau-Pinelli index) and Forced Expiratory Flow (FEF 25-75)] and by evaluation of capillary blood glucose and lactate levels (ABL 800 Flex, Radiometer, Neuilly-Plaisance, France). Exercise sessions were conducted under continuous EKG, respiratory gaz exchange monitoring, SpO_2_, and blood pressure measurements. Exercises were performed 2 h after insulin injection or infusion preceding the previous meal (i.e., lunch) and capillary glucose target levels were set at 70 to 250 mg/dl. Before exercise, hypoglycemia was treated with dextrose (0.3 g/kg body weight) and hyperglycemia (BG >180 mg/dl) was corrected with insulin. However, blood glucose values <50 mg/dl or >300 mg/dl or presence of blood ketones (≥0.6 mM) were considered as contra-indications to exercise.

### Study End Points

The primary study end point was the SG evolution during and after a real-life exercise session after preemptive algorithm-based adaptation of insulin dose (i.e., basal, bolus; reduction, increase) based on SG analysis during and after a first standardized treadmill exercise in pediatric patients with T1D.

### Control Group

A diabetes-free control group was constituted for the cardiac assessment. It consisted of children and adolescents referred to the pediatric cardiology department at Cliniques universitaires Saint-Luc (CUSL) for a standard stress test, an echocardiogram, and a prolonged EKG and for whom these different examinations (as well as individual medical history) did not show signs of heart disease.

### Statistical Analysis

Data were analyzed using the SAS V9.4 software. Categorical variables were analyzed using the chi-square test. The normality of the continuous variables was evaluated using a normal plot and a Q–Q plot. The one-way analysis of variance was applied to compare the averages of the normally distributed variables. The Wilcoxon test was applied when the variable was not distributed normally. On continuous variables, Pearson’s correlation coefficient allowed us to test the linear relationship between two variables. The normality of continuous variables was evaluated using a normal plot and a Q–Q plot pad. The paired sample Student’s *t*-test was used to compare normally distributed averages. For data not following a normal distribution, the Wilcoxon test of signed ranks was used. The statistical significance level used for all analyzes was 0.05. Data are expressed as mean ± SD unless specified.

## Results

### Participants

Our study cohort included 12 adolescents with T1D (aged 11 to 18) and our control cohort consisted of 12 children and adolescents (aged 10 to 17) without history of diabetes or heart disease. These two cohorts were similar in terms of age, gender, and biometrics (height and BMI SD scores) (**Table 1**). In the study cohort, patients were not consuming medications other than insulin (ten under MDI, two under pump therapy) and had levels of diabetes control (mean HbA_1C_ levels: 7.1%) corresponding to average levels achieved in pediatric diabetes conventions of care (16). Patients did not experience episodes of DKA or severe hypoglycemia (grade 3) during the last 5 years before entering the trial. According to PAQ questionnaires, our patients reported mean physical activity levels of 2.1 ± 0.6 that were negatively correlated (*R*: −0.70, *p* = 0.04) with baseline HbA_1C_ levels.

**Table 1:** Characteristics of the clinical series.

	Control group(*N* = 12)	T1D group(*N* = 12)	*p*-value
**Age (years)**			
Mean ± SD	14.7 ± 2.1	15.1 ± 2	.23
Median (min–max)	14 (10–17)	16 (11–18)	
**Height (*Z*-score)**	0.0 ± 0.74	−0.3 ± 0.94	.29
**Weight (*Z*-score)**	0.56 ± 0.67	0.65 ± 0.55	.66
**BMI (*Z*-score)**	0.9 ± 0.85	1.12 ± 0.85	.32
**Sex**			
Girls [% (*n*)]	41.6% (5)	41.6% (5)	.82
Boys [% (*n*)]	53.9% (7)	58.4% (7)	
**Diabetes duration**			
Diabetes duration (years)		9.5 ± 3.1	
5–10 years [% (*n*)]		53.9% (7)	
≥10 years [% (*n*)]		46.2% (6)	
**Glycemic control**			
Basal rate HbA_1C_		7.1 ± 1.2	
≤7% [% (*n*)]		46.2% (6)	
7–8% [% (*n*)]		30.8% (4)	
≥8% [% (*n*)]		23.1% (3)	
Glycemic mean* (md/dl)		156.9 ± 27.5	
Past 3 months hypoglycemia (%)		18.9 ± 6.6	
Insulin doses (IU/day)		64.7 ± 23.9	
Insulin doses (IU/kg/day)		0.99 ± 0.28	
IDAA_1C_**		11.1 ± 1.9	

*Glycemic mean over past 3 months.

**IDAA_1C_: insulin dose-adjusted A_1c,_ has been calculated using the following equation: [4 × insulin dose (UI/kg/day)] + HbA_1c_ (%).

### Standardized and Real-Time Exercise Test

For TE#1, when a resting heart rate was obtained in supine position after at least 5 min of rest, all patients underwent 26.9 ± 3.2 min of moderate-to-vigorous exercise on a treadmill. Resting and peak heart rates (HR, in bpm), baseline and peak systolic and diastolic blood pressures (in mmHg), peak oxygen uptake (VO_2_ max, in ml/kg/min), exercise capacity (METs), and respiratory exchange ratio (RER) are reported in **Tables S1**, **S2**. EKG variables, left ventricular systolic function as assessed by transthoracic echocardiography, and lung function tests obtained before and 3 min after exercise were all within normal limits (**Table S2**). Cardiovascular parameters were similar in the two cohorts, except for values of resting systolic blood pressure and diastolic IVS thickness, both lower in the diabetes cohort than in controls (**Table S2**).

During TE#2, patients exercised for a mean duration of 59.1 ± 21.6 min and crossed a distance of 3.8 ± 1.8 km. The maximal and minimal HR were 164.6 ± 26.9 and 129.4 ± 27.8 bpm, respectively. The estimation of caloric consumption was 438.1 ± 142.4 kcal.

### Insulin Adjustments

Modifications of insulin administration during second sessions of exercise (TE#2) were instituted on either prandrial rapid insulin or basal insulin (i.e., basal rate for pump users [*n* = 2] and long-acting insulin for MDI users [*n* = 10]) scheduled before, during, or up to 15 h after exercise sessions, with both increase and decrease of insulin doses.

Reduction of basal insulin during the night (19:00–07:00) was the most frequently applied change (89%) that corresponded to a decrease of 15% ± 8.9% of the total basal dose (**Table S3**). Decreases of rapid insulin were instituted in 78% of patients (14.2% ± 4.9% of reduction) before exercise and in 22% of patients (15.1% ± 7.1% of reduction) after exercise. Other types of modifications (increase of rapid insulin doses, modifications of daytime basal insulin) were performed more rarely (11% of rapid dose increase [modification of 15% before and 20% after TE]; 11% of basal daytime insulin reduction [reduction of 15% and increase of 20%]).

### SG Evolution During Exercise Sessions

For analysis of SG data, five periods were analyzed: test exercise (TE+0–0.5 h), TE+0.5–4 h (afternoon–evening), TE+4–8 h (evening–nighttime), TE+8–12 h (nighttime), and TE+12–15 h periods (late night). TE#1 sessions occurred between 3:30 and 5:00 p.m. in the course of afternoon snack, which allowed us to evaluate the needs for insulin dose corrections in case of hyperglycemia, or fast-acting sugars intake following hypoglycemia. Real-time TE#2 sessions were performed by patients after the afternoon snack, with a delay of maximum 2 h.

**Figure 1:**
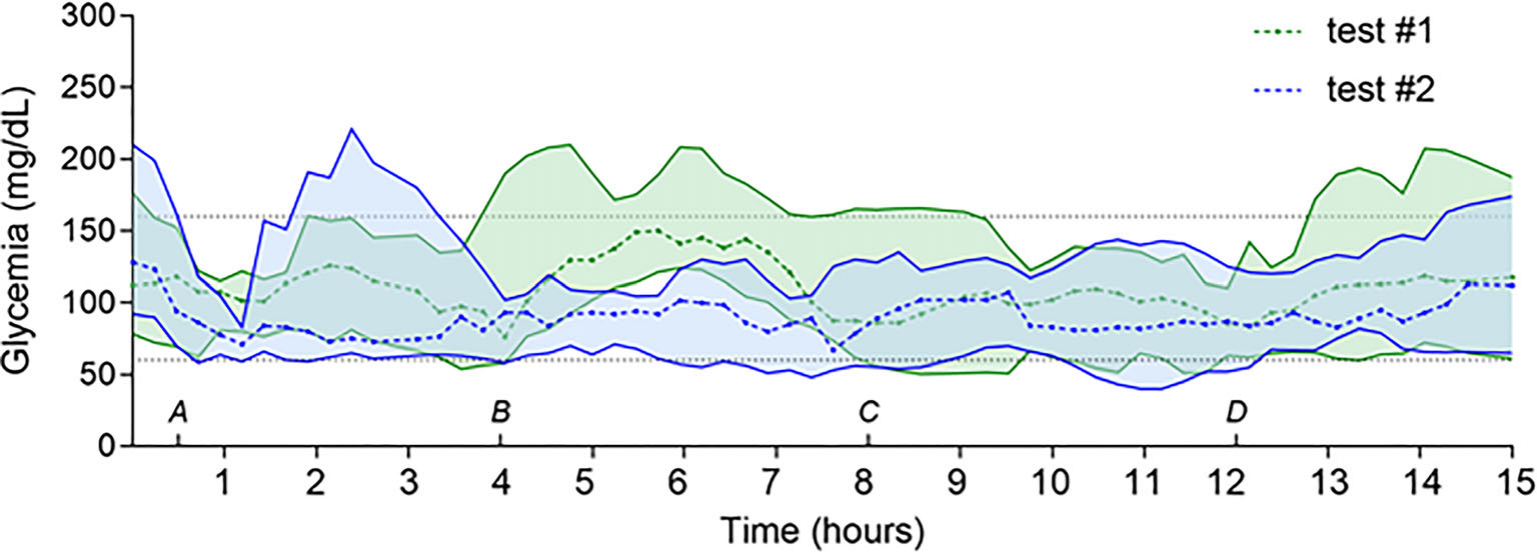
Evolution of SG values during TE#1 and TE#2 in pediatric patients. Legend: Sixteen-hour evaluation of SG in pump and MDI groups during TE#1 (green) and TE#2 (blue) sessions. Gray dashed lines represent minimum (60 mg/dl) and maximum (160 mg/dl) target SG values. **(A–D)** delimitate the different evaluated time points: TE+0–0.5 h (ends at **(A)**, TE+0.5–4 h (afternoon–evening, starts at **(A)**, TE+4–8 h (evening–nighttime, starts at **(B)**, TE+8–12 h (nighttime, starts at **(C)**, and TE+12–15 h periods (starts at **(D)**. Data are expressed as median and interquartile range.

When the 15-h periods were analyzed as a whole, mean SG values improved from 123 ± 63 mg/dl during TE#1 sessions to 104 ± 58 mg/dl during TE#2 sessions (*p* < 0.001) (**Figure 1**). There was a global increase of both time-in-range (defined as SG values between 70 and 179 mg/dl) and higher proportions of hypoglycemia (below 70 and 60 mg/dl) (**Table 2**). Coefficients of variation (CV) were similar between the two sessions (51% and 52% in TE#1 and TE#2, respectively; *p* = 0.28).

**Table 2:** Glucose variations during Test #1 and #2 sessions.

Periods of analysis	Test #1(% of time ± SD)	Test #2(% of time ± SD)	*p*-value
**15 h**			**<0.001**
70–179 mg/dl	54.3 ± 32.6	64.2 ± 30.8	
<70 mg/dl	20.9 ± 14.3	30.9 ± 21.9	
<60 mg/dl	14.5 ± 13.3	19.6 ± 21.6	
≥180 mg/dl	18.2 ± 17.9	9.9 ± 13.9	
**0–0.5 h**			0.26
70–179 mg/dl	62.5 ± 43.3	54.2 ± 49.8	
<70 mg/dl	20.8 ± 33.4	12.5 ± 31.1	
<60 mg/dl	0	12.5 ± 31.1	
≥180 mg/dl	16.7 ± 38.9	33.3 ± 49.2	
**0.5–4 h**			0.16
70–179 mg/dl	65.2 ± 23.5	51.4 ± 28.4	
<70 mg/dl	21.1 ± 20.9	32.9 ± 35.6	
<60 mg/dl	13.2 ± 11.5	17.5 ± 23.3	
≥180 mg/dl	12.7 ± 19.1	16.2 ± 25.6	
**4–8 h**			**<0.001**
70–179 mg/dl	68.7 ± 33.2	65.1 ± 32.3	
<70 mg/dl	8.8 ± 13.4	32.8 ± 31.2	
<60 mg/dl	6.2 ± 10.3	25.0 ± 29.9	
≥180 mg/dl	23.0 ± 33.5	1.9 ± 0.05	
**8–12 h**			**0.015**
70–179 mg/dl	57.8 ± 29.6	67.8 ± 36.8	
<70 mg/dl	29.7 ± 30.7	28.1 ± 38.8	
<60 mg/dl	23.4 ± 28.6	21.9 ± 39.3	
≥180 mg/dl	12.3 ± 22.8	4.4 ± 0.1	
**12–15 h**			0.24
70–179 mg/dl	49.2 ± 40.5	61.7 ± 39.7	
<70 mg/dl	25.8 ± 39.2	28.3 ± 36.9	
<60 mg/dl	18.3 ± 38.6	5.8 ± 14.4	
≥180 mg/dl	25.8 ± 39.6	10 ± 28.9	

Bold is significant (<0.05).

During TE+0–0.5 h and TE+0.5–4 h periods, mean SG tended to decrease in TE#2 as compared to TE#1 (e.g., decrease from 114 ± 52 to 109 ± 64 mg/dl for TE+0.5–4 h; *p* = 0.16). However, SG variability increased with lower percentages of SG values within time-in-range, higher levels of hyperglycemia for TE+0–0.5 h (16.7% ± 38.9% [test #1] to 33.3% ± 49.2% [test #2]) and higher levels of hyper- and hypoglycemia for TE+0.5–4 h (**Table 2**). In TE+0.5–4 h periods, global SG curves during TE#2 were negatively impacted by individual 3-h peaks of hyperglycemia appearing 1 h after exercise, with the resulting effect of increasing CV during the second sessions of tests (58% *vs.* 45% in TE#1, *p* < 0.001).

TE+4–8 h periods of TE#2 were characterized by a marked decrease in mean SG from 144 ± 67 [TE#1] to 93 ± 39 mg/dl [TE#2] (*p* < 0.001), which was mostly due to a net increase of 24% and 18.8% of time spent in hypoglycemia below 70 and 60 mg/dl, respectively, compared to TE#1. CVs did not improve during TE#2 sessions (42% *vs.* 47% in TE#1 sessions), meaning that the SG values were homogeneously reduced, probably due to a net effect of insulin dosage before dinner.

During TE#2 sessions, TE+8–12 h period was marked by lower extents of SG variability (CV: 46% *vs.* 57% during TE#1, *p* < 0.001), improved mean SG levels (from 109 ± 61.2 to 97 ± 43.4 mg/dl; *p* = 0.015), and lower percentages of hypo- and hyperglycemia, compared to the TE#1 sessions. TE+12–15 h periods showed a tendency to similar trends than in TE+8–12 h periods, yet with higher percentages of SG values between 60 and 70 mg/dl so that the global TE+12–15 h periods were marked by the absence of significant improvements of SG values (e.g., mean SG evolved from 83 ± 50 [TE#1] to 78 ± 87 mg/dl [TE#2]; *p* = 0.24). No adverse events (severe hypoglycemia, persistent hyperglycemia, ketosis, or ketoacidosis) occurred during sessions in either group.

Among diabetes-specific clinical variables, we observed that HbA_1C_ levels (at baseline) influenced the participants’ SG values during TE#1 and TE#2 exercise sessions, whereas diabetes duration and insulin dose regimen did not. When we stratified patients in two groups according to HbA_1C_ levels (i.e., <7% and >7%), we obtained a group 1 (<7%) with HbA_1C_ levels at 6.3 ± 0.6% (*n* = 6) and a group 2 (>7%) with HbA_1C_ levels at 7.9 ± 1.1% (*n* = 6). There were substantially lower levels of SG in group 1 during the sessions. Indeed, in groups 1 and 2, SG values corresponded respectively to 109 ± 29 mg/dl and 136 ± 27 mg/dl (*p* < 0.0001) during TE#1, and to 95 ± 31 mg/dl and 113 ± 35 mg/dl (*p* < 0.0001) during TE#2 (**Supplementary Figures 1A, B**). A difference also existed between the two groups while comparing TE#1 and TE#2 sessions: while the delta of mean SG values corresponded to −14 mg/dl from TE#1 to TE#2 (*p* < 0.01) in group 1, this reduction of SG values was higher and corresponded to a delta of −23.6 mg/dl (*p* < 0.0001) in group 2 (**Supplementary Figures 1C, D**). HbA_1C_ levels of study participants did not correlate with mean SG levels during exercise sessions.

### Hypoglycemia

A vast majority of patients experienced hypoglycemia during the day (TE#1: 83%; TE#2: 92% of patients) or the night (TE#1: 75%; TE#2: 75% of patients) following the test exercise, without noticeable differences among groups.

Neither the frequency nor the duration of diurnal and nocturnal hypoglycemia was different among subgroups during the two exercise sessions (frequency of diurnal hypoglycemia: 1.4 ± 0.9 *vs.* 1.1 ± 0.8 episode per day, TE#2 *vs.* TE#1; *p* = 0.05; frequency of nocturnal hypoglycemia: 0.8 ± 0.6 *vs.* 0.9 ± 0.7 episode per day, TE#2 *vs.* TE#1; *p* = 0.36; duration of diurnal hypoglycemia: 97 ± 79 *vs.* 60 ± 58 min per episode, TE#2 *vs.* TE#1; *p* = 0.17; duration of nocturnal hypoglycemia: 152 ± 125 *vs.* 125 ± 86 min per episode, TE#2 *vs.* TE#1; *p* = 0.32).

Also, no differences were noticed in the frequency of hypoglycemia <70 mg/dl during the TE+0.5–4 h, TE+8–12 h, and TE+12–15 h periods (**Table 2**). Yet, during the TE+4–8 h period, mean proportion of hypoglycemia <70 mg/dl rose from 8.8% to 32.8% (TE#1 *vs.* TE#2; *p* = 0.015) and mean proportion of hypoglycemia <60 mg/dl increased from 6.2% to 25% (TE#1 *vs.* TE#2; *p* = 0.036). This discrepancy between proportions and absolute numbers of hypoglycemic events might be explained by the important reduction of mean SG levels (cfr supra) and of the mean percentage of hyperglycemia (from 23 to 1.9%) during this period. Levels of fitness assessed by the PAQ questionnaires did not correlate with the extent or duration of diurnal or nocturnal hypoglycemia.

Recommendations and solutions for fast-acting CHO intake were given to patients during the first exercise sessions, to be used for TE#1 and TE#2. Based on the written documents received after TE#2 sessions, we observed that 55.5% of patients required fast-acting CHO supplements the day before the test, 100% needed extra CHO on the day of TE#2, and 77.8% needed extra CHO the day after the test. The symptoms of hypoglycemia that most adolescents experience were fatigue, weakness, hunger, and dizziness. There was no need for patients to use glucagon injection.

## Discussion

Our CAR2DIAB study, combining standardized exercise and real-life exercise sessions, shows the possibility to improve glycemic control in children and adolescents with T1D using algorithm-based adaptations of insulin doses and carbohydrate intake. In our patient series, fine-tuning of insulin doses allowed an overall normalization in glycemic average and a decrease of hyperglycemia 4 to 15 h after sports. During second exercise sessions, treatment adaptations did not modify the proportions of hypoglycemia, except during a specific post-exercise interval (4 to 8 h) where the rates of hypoglycemia (<60 mg/dl) increased to 25%, yet without the need to stop exercise or activities, and without inducing hypoglycemia-related complications. Our study thus shows that protocols for insulin adjustment and CHO intake reduce the risk for hyperglycemia but are limited in their effectiveness to avoid hypoglycemia, especially in patients under MDI regimens. In our previous TREAD-DIAB study, patients under pump therapy had normalized SG levels (with lower rates of hypoglycemia) during sports when using fine-tuning algorithms for insulin dose modifications. However, during TREAD-DIAB sessions, patients were evaluated during two rounds of identical sports protocols.

Duration and intensity of exercise influence the risk of hypoglycemia, partly because of an increased insulin sensitivity after exercise (2). From 7 to 11 h after exercise, adolescents are likely to require CHO intake to avoid hypoglycemia (17). Late hypoglycemia may occur, especially after a prolonged moderate-to-vigorous physical activity (18). The recurrent pattern of hypoglycemia observed in our study during TE4–8 h is thus congruent with previous findings. Jaggers et al. analyzed the predicting factors of nocturnal hypoglycemia and concluded that adolescents practicing vigorous physical activity are more likely to present prolonged nocturnal hypoglycemia (19). In our study, levels of fitness did not correlate with the risk of experiencing hypoglycemia during sports.

Our CGM analyses showed that mean SG values improved from 123 ± 63 mg/dl during TE#1 to 104 ± 58 mg/dl during TE#2 (*p* < 0.001) with an increase in hypoglycemia. Peaks of hyperglycemia appeared mostly 1 h after exercise in TE#2 (TE0.5–4 h) while SG values decreased significantly during the TE+4–8 h period. Eventually, TE8–12 h and TE12–15 h periods were marked by better SG means and lower percentages of hypo- and hyperglycemia in TE#2 than in TE#1. This means that SG values were homogenously reduced in these periods, which probably resulted from the modified insulin dosage before dinner. This was already underlined in 1985, when Schiffrin and Parikh examined the effects of modifying premeal insulin doses before a 45-min exercise session in 13 adolescents with T1D and found that a reduction of 50% of the premeal dose proved an effective method to reduce the risk of hypoglycemia (20).

During TE#2 sessions, the most frequent insulin dose modification was a reduction of 15% ± 8.9% of basal insulin during nighttime (89%). Rapid insulin reduction of 14.2% ± 4.9% before exercise (78%) and of 15.1% ± 7.1% after exercise (22%) were other frequent adjustments. In our previous TREAD-DIAB study, 82% of CSII patients decreased insulin doses (reduction of 32% ± 12.8%) while a similar reduction (29.4% ± 15.2%) was performed by 65% of MDI patients (9). However, in this series of CSII and MDI patients, we observed that reduction in boluses was more frequent than in basal doses (60% and 68%, respectively). Several protocol divergences may account for these differences, notably the distribution between pump users and MDI users, age of subjects, CHO intake regimen, and type of exercise. In both studies, reductions in insulin doses were comparable and in agreement with literature. The ISPAD recommends, according to the type of physical activity (2), a reduction of 20% to 50% of basal insulin during nighttime for children under CSII and of rapid insulin before exercise for patients under MDI. Since we added carbohydrate intake recommendations in our present study, insulin modifications might have been lower than in the TREAD-DIAB study.

Although all patients had more than 5 years of diabetes duration, no perturbation of cardiac and respiratory function tests could be observed, within limits of the assessment performed (i.e., Resting and peak HRs, baseline and peak systolic and diastolic blood pressures, VO2 max, METs, and RER). Several studies showed that children with T1D presented cardiac anomalies (i.e., Abnormal LV myocardial deformation, LV systolic and diastolic discoordination, and increased peak LV strain rate), in comparison with healthy children, and that these anomalies were associated with diabetes duration (21–23). In our study, values of resting systolic blood pressure and diastolic IVS thickness were lower in the diabetes cohort than in controls. Contrarily, in a recent study, children and adolescents with T1D having echocardiographic signs of reduced diastolic function were shown to have higher blood pressure than controls (24). Further cardiological assessment (including prolonged EKG) would be required to decipher the exact clinical value of these findings.

Our study shows how difficult it is to envision a “one size fits all” protocol for treatment adaptation for patients with T1D (or with complete insulin dependence). For young patients, the challenge is pronounced by the spontaneous and unpredictable nature of exercise, the lack of fitness and the change in metabolism during puberty. Although encouraging, protocols for insulin and CHO adaptation still require refinement to significantly reduce episodes of hypo- and hyperglycemia in children practicing sports, especially when under MDI regimen. We believe that the personalized evaluation of protocols for insulin and CHO adaptations during sports practicing in children and adolescents with T1D should be fully integrated into the clinical follow-up of every patient taken care of in pediatric diabetology centers.

## Conclusion

In our study, the application of algorithmic adaptations of insulin doses and carbohydrate intake in pediatric diabetic patients has globally improved glycemic control during 15 h after real-time exercises performed by children and adolescents with T1D. Because intervention protocols for treatment adaptation during sports will remain influenced by an irreducible level of confounding factors, it is important to envision intervention protocols that introduce the lowest levels of variables and which focus on sports practicing in real-life settings with the aim to propose individualized education and care.

## Data Availability Statement

The raw data supporting the conclusions of this article will be made available by the authors, without undue reservation.

## Ethics Statement

The studies involving human participants were reviewed and approved by Comité d’éthique Hospitalo-Facultaire de l’UCLouvain. Written informed consent to participate in this study was provided by the participants’ legal guardian/next of kin.

## Author Contributions

PL, HA, EG, HB, MO, and SM researched data and wrote the manuscript. TB contributed to the discussion. All authors contributed to the article and approved the submitted version.

## Conflict of Interest

The authors declare that the research was conducted in the absence of any commercial or financial relationships that could be construed as a potential conflict of interest.

## Publisher’s Note

All claims expressed in this article are solely those of the authors and do not necessarily represent those of their affiliated organizations, or those of the publisher, the editors and the reviewers. Any product that may be evaluated in this article, or claim that may be made by its manufacturer, is not guaranteed or endorsed by the publisher.
