# Supplementary material for: Combined Algorithm-Based Adaptations of Insulin Dose and Carbohydrate Intake During Exercise in Children With Type 1 Diabetes: Results From the CAR2DIAB Study
Source: Front Endocrinol (Lausanne). 2021 Aug 26;12:658311. doi: 10.3389/fendo.2021.658311 (PMC8427034; doi:10.3389/fendo.2021.658311)
Supplement: Supplementary file 1 [file DataSheet_1.doc]

**Table S1. Evaluation of the cardiovascular parameters of the clinical series before and during the exercise sessions (part 1).**

**Table S2. Evaluation of the cardiovascular parameters of the clinical series before and during the exercise sessions (part 2).**

**Table S3. Insulin dose modifications during TE#2 sessions.**

**Figure S1: Subgroup analysis of SG evolution in TE#1 and TE#2 sessions.**

**Table S1. Evaluation of the cardiovascular parameters of the clinical series before and during the exercise sessions (part 1).**

| ***Hemodynamic parameters*** | *Control group (N = 12)* | *T1D group (N = 12)* | *P-value* |
| --- | --- | --- | --- |
| **Rest** | | | |
| HR (bpm) | 82.3 ± 13.4 | 83.9 ± 19.7 | .81 |
| Systolic BP (mmHg) | 118.1 ± 9.6 | 115.2 ± 14.3 | .56 |
| Diastolic BP (mmHg) | 62.4 ± 9.5 | 65.3 ± 10.5 | .47 |
| **Maximal effort** | | | |
| HR (bpm) | 193.1 ± 6.0 | 194.7 ± 9.1 | .59 |
| Maximal HR based on age (%) | 93 ± 2.6 | 94.3 ± 4.6 | .38 |
| Systolic BP (mmHg) | 162.5 ± 16.5 | 171.7 ± 28.6 | .33 |
| Diastolic BP (mmHg) | 54 ± 12.1 | 60 ± 18.2 | .34 |
| **Recovery** | | | |
| Systolic BP (mmHg) | 133.4 ± 12.5 | 119 ± 16.4 | **.02** |
| Diastolic BP (mmHg) | 56.9 ± 11.3 | 58.8 ± 12.9 | .69 |
| **Difference between parameters during maximal effort and at rest** | | | |
| Maximal systolic BP – Systolic BP at rest | 44.4 ± 19.3 | 56.4 ± 19.4 | .13 |
| Maximal diastolic BP – Diastolic BP at rest | -8.38 ± 12.3 | -5.3 ± 14.3 | .57 |

**Table S2. Evaluation of the cardiovascular parameters of the clinical series before and during the exercise sessions (part 2).**

|  | *Control group (N = 12)* | *T1D group (N = 12)* | *P-value* |
| --- | --- | --- | --- |
| **Electrocardiogram** | | | |
| PR (ms) | 132.1 ± 14.8 | 139.0 ± 16.5 | .24 |
| QTc (ms) | 414.9 ± 19.8 | 417.2 ± 32.5 | .13 |
| **Echocardiography** | | | |
| Diameter of the left ventricle in diastole (cm) | 4.6 ± 0.3 | 4.8 ± 0.4 | .24 |
| Wall thickness of the left ventricle in diastole (cm) | 0.72 ± 0.09 | 0.65 ± 0.10 | .09 |
| Thickness IVS in diastole (cm) | 0.82 ± 0.1 | 0.67 ± 0.13 | **.003** |
| Ejection fraction (%) | 65.8 ± 6.5 | 61.4 ± 8.5 | .16 |
| Shortening fraction (%) | 36.5 ± 5.5 | 34.4 ± 5.3 | .37 |
| **Performance** | | | |
| V_O2_ max (mL/kg/min) | 43.6 ± 9.7 | 37.9 ± 8.5 | .13 |
| V_O2_ max (% moyenne) | 105.9 ± 22.8 | 91.7 ± 14.3 | .08 |
| Maximal load (METs) | 14.1 ± 3.3 | 12.9 ± 2.1 | .27 |
| Anaerobic threshold (min) | 10.1 ± 2.6 | 9.8 ± 2.9 | .84 |
| Anaerobic threshold (% of theoretical V_O2_ max) | 72.3 ± 12.1 | 66.8 ± 7.7 | .24 |
| Maximum respiratory quotient | 1.4 ± 0.2 | 1.3 ± 0.2 | .10 |

**Table S3. Insulin dose modifications during TE#2 sessions.**

| **Insulin type** | **Timing of modification** |  | **Type of modification** | |
| --- | --- | --- | --- | --- |
|  |  |  | **Reduction** | **Increase** |
| Rapid | Before TE | *n* | 7 | 1 |
|  |  | Dose (%, mean ± SD) | 14.2 ± 4.9 | 15 |
|  |  |  |  |  |
|  | After TE | *n* | 2 | 1 |
|  |  | Dose (%, mean ± SD) | 15.1 ± 7.1 | 20 |
|  |  |  |  |  |
| Basal | Daytime | *n* | 1 | 1 |
|  |  | Dose (%, mean ± SD) | 15 | 20 |
|  |  |  |  |  |
|  | Nighttime | *n* | 8 | 0 |
|  |  | Dose (%, mean ± SD) | 15 ± 8.9 |  |

**Figure Legends**

**Figure S1. Subgroup analysis of SG evolution in TE#1 and TE#2 sessions.**

Legend: Graphs show the evolution of SG values in patients according to their levels of HbA_1C_ at baseline (i.e. <7% or >7%). (**A**): SG evolution during TE#1 sessions in patients with HbA_1C_ <7% (magenta) and with HbA_1C_ >7% (orange). (**B**): SG evolution during TE#2 sessions in patients with HbA_1C_ <7% (magenta) and with HbA_1C_ >7% (orange). (**C**): SG evolution during TE#1 (green) and TE#2 (blue) sessions in patients with HbA_1C_ <7% (magenta dots). (**D**): SG evolution during TE#1 (green) and TE#2 (blue) sessions in patients with HbA_1C_ >7% (orange dots). Grey dashed lines represent minimum (60 mg/dL) and maximum (160 mg/dL) target SG values. A, B, C, and D delimitates the different evaluated time points: TE+0-0.5h (ends at A), TE+0.5-4h (afternoon-evening, starts at A), TE+4-8h (evening-nighttime, starts at B), TE+8-12h (nighttime, starts at C) and TE+12-15h periods (starts at D). Data are expressed as median and interquartile range.
