# Supplementary figures and images for: Combined Algorithm-Based Adaptations of Insulin Dose and Carbohydrate Intake During Exercise in Children With Type 1 Diabetes: Results From the CAR2DIAB Study
Source: Front Endocrinol (Lausanne). 2021 Aug 26;12:658311. doi: 10.3389/fendo.2021.658311 (PMC8427034; doi:10.3389/fendo.2021.658311)

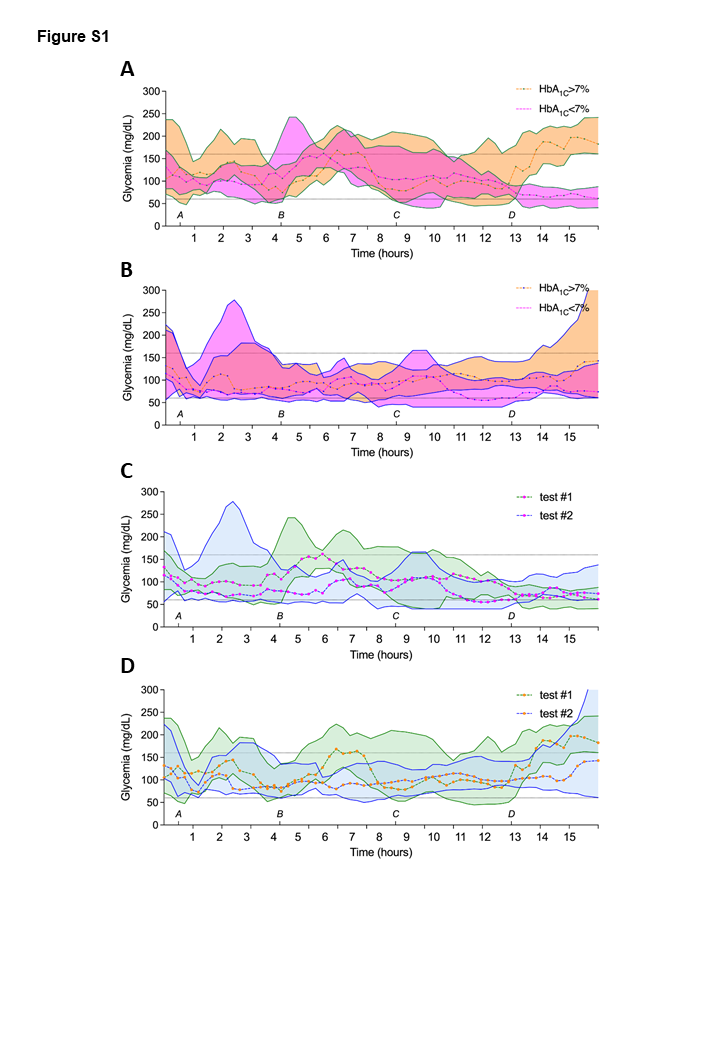

Supplement: Supplementary file 2 [file Image_1.tif]
